# Nashville Journal of Medicine and Surgery

**Published:** 1851-08

**Authors:** 


*Nashville Journal of Medicine and Surgery.*—This is the title of a new monthly periodical of which the two first numbers have been received. It is issued at Nashville, Tennessee, under the editorial management of W. K. Boling, M. D., Prof. of the Institutes and Practice of Medicine in the Med. Department of the University of Nashville. The numbers that have appeared give promise of merit and success.

